# Prognostic value of early post-treatment ^18^F-FDG PET/CT in diffuse large B-cell lymphoma patients receiving chimeric antigen receptor T-cell therapy

**DOI:** 10.1186/s40644-025-00888-8

**Published:** 2025-06-08

**Authors:** Seyed Ali Mirshahvalad, Andres Kohan, Roshini Kulanthaivelu, Claudia Ortega, Ur Metser, David Hodgson, Robert Kridel, Christine Chen, Sita Bhella, Kelly Yuen Wai Chin, Patrick Veit-Haibach

**Affiliations:** 1 Joint Department of Medical Imaging, University Medical Imaging Toronto (UMIT), University Health Network, Mount Sinai Hospital & Women’s College Hospital, University of Toronto, Toronto, ON Canada; 2 Medical Oncology and Hematology, Princess Margaret Cancer Centre, University Health Network, Toronto, ON Canada

**Keywords:** Fluorodeoxyglucose, Positron emission tomography, Lymphoma, Chimeric antigen receptor, Prognosis, Survival

## Abstract

**Purpose:**

To evaluate the prognostic value of early post-treatment ^18^F-FDG PET/CT in diffuse large B-cell lymphoma (DLBCL) patients undergoing chimeric antigen receptor T-cell (CAR-T) therapy.

**Methods:**

In this retrospective study, 159 patients referred for imaging prior to CAR-T therapy between January 2018 and May 2023 were reviewed. Of those, 51 with both baseline pre-infusion and one-month post-treatment ^18^F-FDG PET/CTs were included. ^18^F-FDG PET/CT parameters were derived, including standard uptake values (SUVs), metabolic tumour volume (MTV), total lesion glycolysis (TLG), and Dmax. Additionally, the delta changes from the baseline were calculated. Time to progression/death was documented. For progression-free survival (PFS) and overall survival (OS), univariate analysis was performed using the Kaplan-Meier method. The significance of the difference was measured using the Mantel-Cox log-rank test. Significant parameters entered the multiple Cox regression.

**Results:**

Overall, 51 patients (mean age = 56y) entered the study. All had Deauville scores of 4 (14/51; 28%) or 5 (37/51; 72%) at baseline. At one month, 28% of patients showed a complete metabolic response, while 72% had ^18^F-FDG-avid significant residual disease. Investigating those with residual disease, SUVmax, SUVpeak, SUVmax-to-Liver ratio and MTV were significantly lower in the one-month post-treatment scan. For PFS evaluation, serum LDH, one-month post-treatment SUVmax-to-liver ratio, one-month post-treatment TLG, and baseline Dmax entered the multivariate analysis. The one-month post-treatment SUVmax-to-liver ratio (Hazard ratio [HR] = 5.21; *p* = 0.004) and baseline Dmax (HR = 13.8; *p* = 0.013) retained significance, being independent predictors of PFS. For OS, serum LDH, delta SUVmean-to-liver ratio, delta percentage TLG, and one-month post-treatment Dmax were included in the multivariate analysis. The delta percentage TLG (HR = 4.37; *p* = 0.023) remained significant as an independent predictor of OS.

**Conclusion:**

Early post-treatment ^18^F-FDG PET/CT can provide valuable prognostic information for DLBCL patients receiving CAR-T. The most significant predictors of outcomes would be the baseline extent of the disease, one-month post-treatment avidity, and changes in the metabolic burden from baseline.

## Introduction

Comprising nearly one-third of non-Hodgkin’s lymphoma, diffuse large B-cell lymphoma (DLBCL) is considered the most common lymphoid malignancy [1, 2]. The standard of care treatment for patients with DLBCL is chemo-immunotherapy [3]. Although effective, nearly 50% of patients potentially suffer from relapse and either receive another line of systemic therapy or undergo autologous stem cell transplantation. Additionally, the survival of patients with relapsed/refractory DLBCL is poor, having an overall survival (OS) of no longer than six months in most cases [4]. Hence, to improve outcomes, the administration of chimeric antigen receptor T-cell (CAR-T) was approved for DLBCL treatment in cases that experienced failure of two or more lines of systemic therapy. This therapy achieves promising, durable responses in a significant proportion of patients with relapsed/refractory DLCBCL [2].

^18^F-FDG positron emission tomography/computed tomography (PET/CT) is considered a highly informative modality for evaluating lymphoma patients, being the current standard of care in a wide range of clinical applications [5, 6]. In the context of CAR-T therapy, ^18^F-FDG PET/CT has been investigated in relapsed/refractory DLBCL and reported to be potentially capable of predicting treatment success, predicting the probability of adverse events occurrence and was also shown to be independently correlated with OS [4, 7–9].

There is, however, not much literature available about the values of PET/CT in the post-therapy setting. It was suggested that categorizing patients into responsive versus non-responsive groups based on the early one-month post-treatment ^18^F-FDG PET/CT could be of value since this may predict their further prognosis, being a more robust assessment compared to the later three-month imaging [10]. While one-month post-treatment complete metabolic responders have favourable long-term outcomes, approximately one-third of patients with residual disease may suffer from early relapse [11]. Furthermore, studies investigating ^18^F-FDG PET/CT-derived metabolic parameters have shown that high one-month post-treatment SUVmax and MTV in patients would be indicative of poorer survival [12–15].

The goal of this study was to determine the benefits of early post-treatment assessment by aiming to evaluate the prognostic value of one-month post-treatment ^18^F-FDG PET/CT in DLBCL patients undergoing CAR-T therapy. In addition, we evaluated the combined value of ^18^F-FDG PET/CT and clinical variables to provide a more robust patient prognostication.

## Materials and methods

### Patient population and studied parameters

**Fig. 1:**
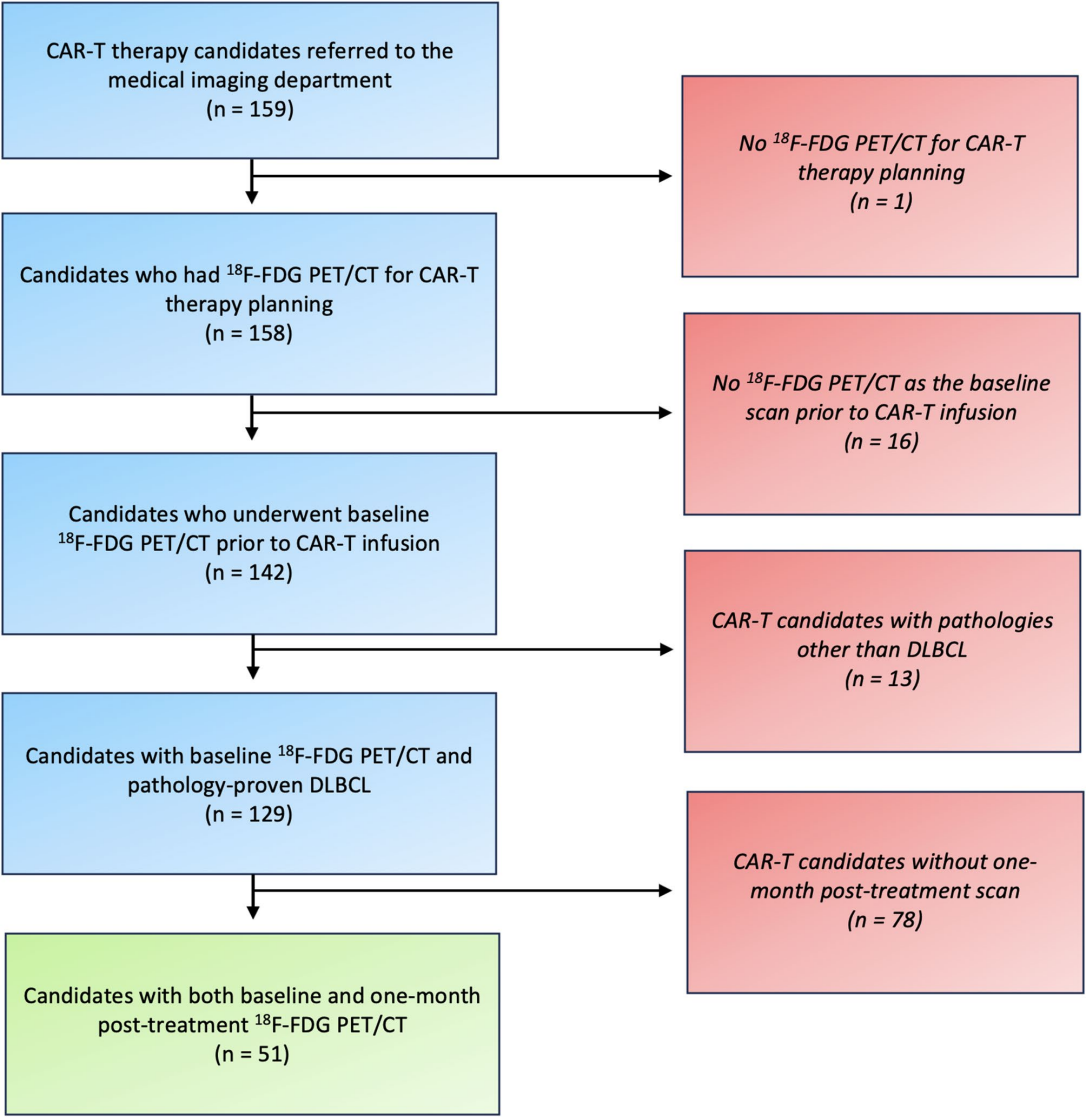
Patient selection flowchart

This retrospective study received approval from the ethics committee and institutional review board (#23-5414). We reviewed 159 patients referred to our department to undergo imaging prior to receiving CAR-T therapy between January 2018 and May 2023. The eligibility criteria to enter this study were patients with one-month post-CAR-T ^18^F-FDG PET/CT who had histopathology-proven DLBCL. All patients were diagnosed with relapsed/refractory DLBCL (with failure of at least two lines of standard therapies) and had baseline pre-infusion ^18^F-FDG PET/CT scans as well. Finally, 51 patients fulfilled the inclusion criteria and entered the current study. Figure 1 shows the patient selection flowchart. Demographic details (e.g., sex, age, race), clinical information (e.g., Lugano stage, ECOG score, serum LDH level, previous treatments, history of treatment failures) and histopathology details of patients were gathered. Additionally, international prognostic indices at baseline were determined, including the International Prognostic Index (IPI), its NCCN version (NCCN-IPI) and the more recently introduced International Metabolic Prognostic Index (integrating age, stage and MTV) [16].

### ^18^F-FDG PET/CT imaging protocol

^18^F-FDG-PET/CT imaging was conducted based on our standardized institutional protocol. Imaging was performed in 3D mode with a dedicated in-line PET/CT scanner (Siemens Biograph mCT 40, Siemens Healthineers, Erlangen, Germany). Patients were instructed to fast for at least 6 h before the radiopharmaceutical administration. ^18^F-FDG was then injected intravenously (approximately 5 MBq/Kg of body weight). A spiral CT imaging was performed from the skull base to the upper thighs (whole-body imaging) unless there was a documented/suspicious involvement of the lower limbs, leading to total-body (true whole-body) acquisition. The scan was performed with the following parameters: 120 kV peak, 40 to 105 mAs, 3.0 mm slice width, 2.0 mm collimation, an overlap of 2.0 mm, 0.8 s rotation time and 8.4 mm feed/rotation. Following CT, PET images were acquired 60 min after ^18^F-FDG administration for 3 min/bed position. A PET scan using scatter correction was obtained, covering the field of view of the CT acquisition. The PET image dimensions were a pixel size of 2.6 × 2.6 mm and a slice thickness of 3.27 mm, filtered with 4-mm full width at half maximum Gaussian filter.

### ^18^F-FDG PET/CT interpretation and image analysis

^18^F-FDG PET/CT scans were interpreted by two expert physicians in consensus using a standard imaging workstation (Mirada XD Workstation, Mirada Medical). ^18^F-FDG-avid lymphoma manifestations were identified and documented. Deauville scores were determined based on the relative uptake of the hottest manifestations compared to the mediastinal and hepatic backgrounds. Deauville scores of 4–5 were considered a significant residual disease in the one-month post-treatment scan. From ^18^F-FDG PET/CTs, SUVmax, SUVmean, SUVpeak (1-mm spherical VOI), whole-body MTV and whole-body TLG were extracted. Notably, MTV was calculated with 41% SUVmax thresholding [17]. Also, hepatic uptake was considered to calculate tumour-to-liver background ratios using 3 cm^3^ VOI in the noninvolved right hepatic lobe. Regarding the distance of tumoral involvement, the furthest distance between tumoral lesions throughout the body (Dmax) and their maximum distance from the spleen (spleen Dmax) were calculated. These distances were also normalized based on body surface area ($\:\sqrt{(weight\:x\:height/3600}$) to generate standardized Dmax and standardized Spleen Dmax [18, 19]. Lastly, the changes in these parameters from the pre-infusion PET/CT were also calculated.

### Patient follow-up

All patients were followed up until July 31, 2024. The date and location of the disease progression were recorded. Also, in case of patient death, the date was documented. Time to progression/death was calculated from the CAR-T administration to the documented progression/death or the final follow-up.

### Statistical analysis

Continuous and categorical parameters were presented as mean ± standard deviation (SD) and frequency (%), respectively. The head-to-head comparison of the ^18^F-FDG PET/CT parameters in the baseline versus one-month post-treatment scan was performed using a paired t-test. Regarding prognostic evaluation, the continuous variables were converted into dichotomized variables using receiver-operating characteristic (ROC) curve analysis and taking the coordinate point with the highest Youden index as the cut-off. For PFS and OS prognostication, univariate analysis was performed using the Kaplan-Meier method. The significance of the difference was measured using the univariate Mantel-Cox log-rank test. Significant parameters in the univariate analysis entered the multiple Cox regression and were presented with their hazard ratio (HR) along a 95% confidence interval (95%CI). All dichotomized versions of the variables showed better prognostication value compared to their continuous counterparts. Thus, only dichotomized variables were considered for multivariate analysis. Moreover, among ^18^F-FDG PET/CT-derived variables with high collinearity (three main groups: “SUVs”, “MTV/TLG”, and “Distances”), we selected parameters with the highest prognostication (based on stepwise variable selection). Moreover, between the three groups of “baseline”, “one-month post-treatment”, and “delta” measurements, we, again, selected the most prognostic parameter to enter multivariate analysis. Notably, no missing data needing treatment was present in the database. All data were analyzed using SPSS software (IBM SPSS Statistics, version 29, IBM Corp., NY, USA). The statistical significance level was set at a two-sided *p*-value less than 0.05.

## Results

Overall, 51 patients (mean age = 56 years) with pathology-proven DLBCL who underwent both pre-CAR-T and one-month post-treatment ^18^F-FDG PET/CTs entered the study. The patient selection flowchart is provided in Fig. 1. Detailed characteristics of the study population can be found in Table 1.

**Table 1:** Detailed characteristics of the studied patient population

	Mean (± SD)/ Frequency (%)
**Age at CAR-T (years)**	56.1 (± 12.6)
**Weight at CAR-T (kg)**	74.5 (± 20.7)
**BMI at CAR-T (kg/m**^**2**^)	25.6 (± 5.9)
**Sex**	
- **Female** - **Male**	19 (37%) 32 (63%)
**Race**	
- **White** - **Asian** - **Other**	38 (74%) 8 (16%) 5 (10%)
**Initial stage**	
- **I** - **II** - **III** - **IV**	1 (2%) 6 (12%) 9 (18%) 35 (68%)
**Number of relapses (episodes of refractory treatment prior to CAR-T)**	
- **2** - **3** - **4** - **5**	36 (71%) 14 (27%) 0 (0%) 1 (2%)
**Pre-CAR-T stage**	
- **I** - **II** - **III** - **IV**	1 (2%) 7 (14%) 4 (8%) 39 (76%)
**Serum LDH level at CAR-T (Normal range: 125–220 U/L)**	341.5 (± 249.2)

Patients underwent pre-infusion and one-month post-treatment ^18^F-FDG PET/CTs with mean intervals of 11 (± 5; median: 10) and 33 (± 8; median: 31) days prior to and after CAR-T infusion, respectively. All patients had Deauville scores of 4 (14/51; 28%) or 5 (37/51; 72%) at baseline. In the one-month post-treatment scans, 14 (28%) patients showed a complete metabolic response to treatment. The other 37/51 (72%) were positive for an ^18^F-FDG-avid significant residual disease, having Deauville scores of 4 (20/37; 54%) and 5 (17/37; 46%). ^18^F-FDG PET/CT-derived measurements of the positive post-treatment scans with residual disease (*n* = 37) are presented in Table 2. Additionally, baseline measurements and changes (delta) from baseline scan to one-month post-treatment are provided.

**Table 2:** One-month post-treatment versus baseline ^18^F-FDG PET/CT-derived parameters in patients with residual disease one month after CAR-T infusion (*n* = 37). Also, the delta changes (between the one-month scan and baseline) are provided

	Mean (± SD)	Baseline vs. one-month scan (*p*-value)
SUVmax		0.010*
- Baseline - Post-treatment - Delta [percentage]	20.93 (± 9.42) 15.63 (± 11.26) -18.55% (± 55.88)	
SUVmean		0.386
- Baseline - Post-treatment - Delta [percentage]	6.78 (± 2.14) 7.54 (± 5.50) + 14.60 (± 78.33%)	
SUVpeak		< 0.001*
- Baseline - Post-treatment - Delta [percentage]	19.09 (± 8.57) 11.40 (± 8.49) -33.96% (± 50.26%)	
Liver Background SUVmean		0.667
- Baseline - Post-treatment - Delta [percentage]	2.22 (± 0.38) 2.18 (± 0.57) + 2.31% (± 17.17%)	
SUVmax-to-Liver ratio		0.013*
- Baseline - Post-treatment - Delta [percentage]	9.70 (± 4.78) 7.31 (± 5.52) -17.60% (± 61.51%)	
SUVmean-to-Liver ratio		0.370
- Baseline - Post-treatment - Delta [percentage]	3.12 (± 1.18) 3.50 (± 2.55) + 17.24% (± 87.30%)	
MTV		0.025*
- Baseline - Post-treatment - Delta [percentage]	228.76 (± 320.39) 105.33 (± 181.51) -63.11% (± 342.05%)	
TLG		0.195
- Baseline - Post-treatment - Delta [percentage]	1721.31 (± 2921.23) 996.54 (± 2097.49) -55.83 (± 304.93%)	
Dmax (cm)		0.490
- Baseline - Post-treatment - Delta [percentage]	30.55 (± 22.69) 27.53 (± 31.35) -4.08% (104.32%)	
Standardized Dmax (cm^− 1^)		0.396
- Baseline - Post-treatment - Delta [percentage]	16.88 (± 12.87) 14.46 (± 15.86) -4.08% (104.32%)	
SDmax (Spleen Dmax) (cm)		0.269
- Baseline - Post-treatment - Delta [percentage]	30.59 (± 17.18) 28.21 (± 14.22) -0.43% (41.64%)	
Standardized SDmax (cm^− 1^)		0.266
- Baseline - Post-treatment - Delta [percentage]	16.55 (± 8.41) 15.31 (± 7.35) -0.43% (41.64%)	

**Fig. 2:**
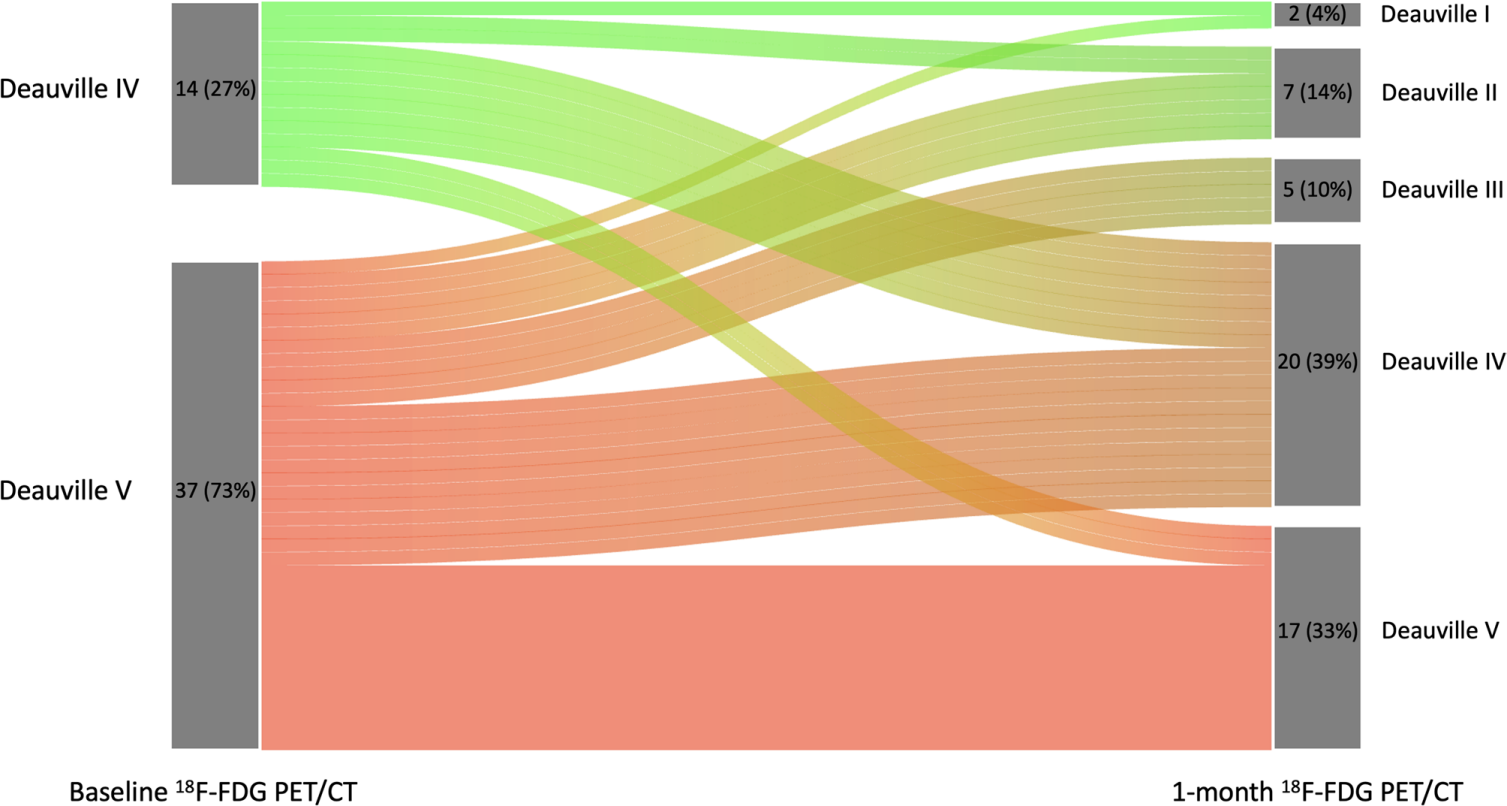
Disease burden changes between the baseline pre-infusion and one-month post-treatment ^18^F-FDG PET/CTs (*n* = 51)

As seen, SUVmax, SUVpeak, SUVmax-to-Liver ratio and MTV were significantly lower in the one-month post-treatment scan compared to the baseline ^18^F-FDG PET/CT. Furthermore, the Deauville score changes from the pre-infusion to one-month post-treatment ^18^F-FDG PET/CTs are shown in Fig. 2.

In the survival analyses, the correlation of the one-month post-treatment ^18^F-FDG PET/CT-derived parameters with PFS and OS was evaluated. Also, the value of the delta changes compared to the pre-infusion was assessed. For all 51 patients, the median PFS and OS were 174 and 356 days, respectively. In patients (37/51) with significant residual disease on one-month post-treatment ^18^F-FDG PET/CT, these were 150 and 301 days, respectively. Notably, 21/51 (41%) deaths were documented in the follow-up.

Details of the univariate analysis for PFS and OS can be found in Tables 3, 4 and 5–6, respectively.

**Table 3:** Detailed results of the progression-free survival univariate analysis

	Hazard Ratio	*p*-value
Sex		0.998
High Age (> 60)		0.288
High BMI (> 24)		0.196
Abnormal LDH (> 220)	2.852 (1.077–7.556)	0.035*
High LDH (> 250)	3.572 (1.349–9.455)	0.010*
Stage III/IV at CAR-T		0.411
High IPI (> 2)		0.333
High NCCN-IPI (> 3)		0.333
High IMPI Risk (> 12)		0.063
High SUVmax (> 15)	2.973 (1.026–8.613)	0.045*
High SUVmean (> 5)		0.553
High SUVpeak (> 10)		0.069
High SUVmax-to-Liver ratio (> 5)	4.557 (1.077–19.279)	0.039*
High SUVmean-to-Liver ratio (> 3)		0.099
High MTV (> 20)	4.302 (1.291–14.334)	0.018*
High TLG (> 80)	5.427 (1.281–22.982)	0.022*
High Dmax (> 14)	10.664 (1.442–78.882)	0.020*
High Standardized Dmax (> 8)	4.947 (1.485–16.479)	0.009*
High Spleen Dmax (> 23)		0.708
High Standardized Spleen Dmax (> 16)		0.304
One-month post-treatment CR		0.096
One-month post-treatment High SUVmax (> 10)	4.767 (2.148–10.579)	< 0.001*
One-month post-treatment High SUVmean (> 4)	4.277 (1.851–9.883)	< 0.001*
One-month post-treatment High SUVpeak (> 7)	4.767 (2.148–10.579)	< 0.001*
One-month post-treatment High SUVmax-to-Liver ratio (> 3)	5.985 (2.246–15.952)	< 0.001*
One-month post-treatment High SUVmean-to-Liver ratio (> 2)	5.578 (2.332–13.341)	< 0.001*
One-month post-treatment High MTV (> 45)	4.624 (2.086–10.254)	< 0.001*
One-month post-treatment High TLG (> 500)	10.644 (4.058–27.916)	< 0.001*
One-month post-treatment High Dmax (> 12)	5.287 (2.427–11.519)	< 0.001*
One-month post-treatment High Standardized Dmax (> 7)	5.287 (2.427–11.519)	< 0.001*
One-month post-treatment High Spleen Dmax (> 25)	3.273 (1.516–7.069)	0.003*
One-month post-treatment High Standardized Spleen Dmax (> 16)	3.351 (1.564–7.182)	0.002*
Delta SUVmax [crude] (-1<)	3.286 (1.511–7.149)	0.003*
Delta SUVmax [percentage] (increase/less than 50% decrease)	3.223 (1.439–7.220)	0.004*
Delta SUVmean [crude] (1<)	5.441 (2.418–12.244)	< 0.001*
Delta SUVmean [percentage] (increase/less than 30% decrease)	4.130 (1.789–9.532)	< 0.001*
Delta SUVpeak [crude] (-2<)	3.035 (1.312–7.018)	0.009*
Delta SUVpeak [percentage] (increase/less than 50% decrease)	3.649 (1.585–8.398)	0.002*
Delta SUVmax-to-Liver ratio [crude] (-1<)	7.319 (3.102–17.270)	< 0.001*
Delta SUVmax-to-Liver ratio [percentage] (increase/less than 50% decrease)	3.530 (1.535–8.120)	0.003*
Delta SUVmean-to-Liver ratio [crude] (-1<)	37.469 (9.243-151.888)	< 0.001*
Delta SUVmean-to-Liver ratio [percentage] (increase/less than 30% decrease)	3.821 (1.608–9.082)	0.002*
Delta MTV [crude] (increase)		0.877
Delta MTV [percentage] (more than 100% increase)	3.512 (1.384–8.914)	0.008*
Delta TLG [crude] (-60<)		0.610
Delta TLG [percentage] (increase/less than 50% decrease)		0.052
Delta Dmax [crude] (3<)	3.862 (1.645–9.067)	0.002*
Delta Standardized Dmax [crude] (1<)	3.862 (1.645–9.067)	0.002*
Delta Dmax [percentage] (increase/less than 10% decrease)	3.109 (1.422–6.796)	0.004*
Delta Spleen Dmax [crude] (7<)	6.742 (2.481–18.319)	< 0.001*
Delta Standardized Spleen Dmax [crude] (1<)	2.392 (1.041–5.497)	0.040*
Delta Spleen Dmax [percentage] (increase/less than 30% decrease)		0.052

*significant

**Table 4:** Detailed results of the progression-free survival multivariate analysis

	Hazard Ratio	*p*-value
High LDH (> 250)	1.499 (0.522–4.311)	0.452
High one-month post-treatment SUVmax-to-liver ratio (> 3)	5.213 (1.715–15.846)	0.004*
High one-month post-treatment TLG (> 100)	2.248 (0.868–5.822)	0.095
High baseline Dmax (> 14)	13.076 (1.702-100.491)	0.013*

*significant

**Table 5:** Overall survival univariate analysis’ detailed results

	Hazard Ratio	*p*-value
Sex		0.678
High Age (> 60)		0.637
High BMI (> 30)		0.109
Abnormal LDH (> 220)	5.778 (1.343–24.865)	0.018*
High LDH (> 250)	5.551 (1.632–18.885)	0.006*
Stage III/IV at CAR-T		0.797
High IPI (> 2)		0.148
High NCCN-IPI (> 3)		0.148
High IMPI Risk (> 12)		0.078
High SUVmax (> 10)		0.176
High SUVmean (> 5)		0.249
High SUVpeak (> 10)		0.139
High SUVmax-to-Liver ratio (> 5)		0.159
High SUVmean-to-Liver ratio (> 2)		0.141
High MTV (> 110)		0.109
High TLG (> 250)		0.078
High Dmax (> 22)		0.101
High Standardized Dmax (> 8)		0.079
High Spleen Dmax (> 25)		0.129
High Standardized Spleen Dmax (> 12)		0.220
One-month post-treatment CR		0.646
One-month post-treatment High SUVmax (> 10)	3.313 (1.196–9.182)	0.021*
One-month post-treatment High SUVmean (> 5)		0.050
One-month post-treatment High SUVpeak (> 5)		0.066
One-month post-treatment High SUVmax-to-Liver ratio (> 3)		0.066
One-month post-treatment High SUVmean-to-Liver ratio (> 2)		0.085
One-month post-treatment High MTV (> 350)	4.558 (1.256–16.542)	0.021*
One-month post-treatment High TLG (> 500)		0.107
One-month post-treatment High Dmax (> 60)	3.754 (1.129–12.481)	0.031*
One-month post-treatment High Standardized Dmax (> 28)		0.053
One-month post-treatment High Spleen Dmax (> 30)		0.203
One-month post-treatment High Standardized Spleen Dmax (> 18)		0.147
Delta SUVmax [crude] (-5<)	3.313 (1.196–9.182)	0.021*
Delta SUVmax [percentage] (more than 10% increase)	3.477 (1.129–10.705)	0.030*
Delta SUVmean [crude] (-5<)	3.885 (1.076–14.023)	0.038*
Delta SUVmean [percentage] (increase/less than 20% decrease)	4.158 (1.290-13.405)	0.017*
Delta SUVpeak [crude] (-2<)		0.051
Delta SUVpeak [percentage] (increase/less than 50% decrease)	2.891 (1.008–8.289)	0.048*
Delta SUVmax-to-Liver ratio [crude] (0<)	4.730 (1.486–15.058)	0.009*
Delta SUVmax-to-Liver ratio [percentage] (increase)	4.730 (1.486–15.058)	0.009*
Delta SUVmean-to-Liver ratio [crude] (0<)	5.065 (1.525–16.818)	0.008*
Delta SUVmean-to-Liver ratio [percentage] (increase)	5.065 (1.525–16.818)	0.008*
Delta MTV [crude] (-10<)		0.339
Delta MTV [percentage] (increase/less than 50% decrease)	3.311 (1.172–9.355)	0.024*
Delta TLG [crude] (-100<)		0.339
Delta TLG [percentage] (increase/less than 50% decrease)	5.709 (1.764–18.478)	0.004*
Delta Dmax [crude] (0<)		0.213
Delta Standardized Dmax [crude] (0<)		0.213
Delta Dmax [percentage] (increase/less than 30% decrease)	3.569 (1.317–9.676)	0.012*
Delta Spleen Dmax [crude] (-7<)		0.793
Delta Standardized Spleen Dmax [crude] (-3<)		0.529
Delta Spleen Dmax [percentage] (increase/less than 30% decrease)	2.778 (1.015–7.601)	0.047*

*significant

**Table 6:** Detailed results of the overall survival multivariate analysis

	Hazard Ratio	*p*-value
High LDH (> 250)		0.534
High delta SUVmean-to-liver ratio (> 0)		0.501
High delta percentage TLG (increase/less than 50% decrease)	4.366 (1.224–15.582)	0.023*
High one-month post-treatment Dmax (> 60)		0.223

*significant

### Progression-free survival

Univariate analysis results can be found in Table 3. From the significant variables and after removing high-collinear parameters, we entered the following categorical parameters into the multivariate Cox analysis (Table 4): serum LDH (cut-off = 250), one-month post-treatment SUVmax-to-liver ratio (cut-off = 3), one-month post-treatment TLG (cut-off = 100), and baseline Dmax (cut-off = 14).

**Fig. 3:**
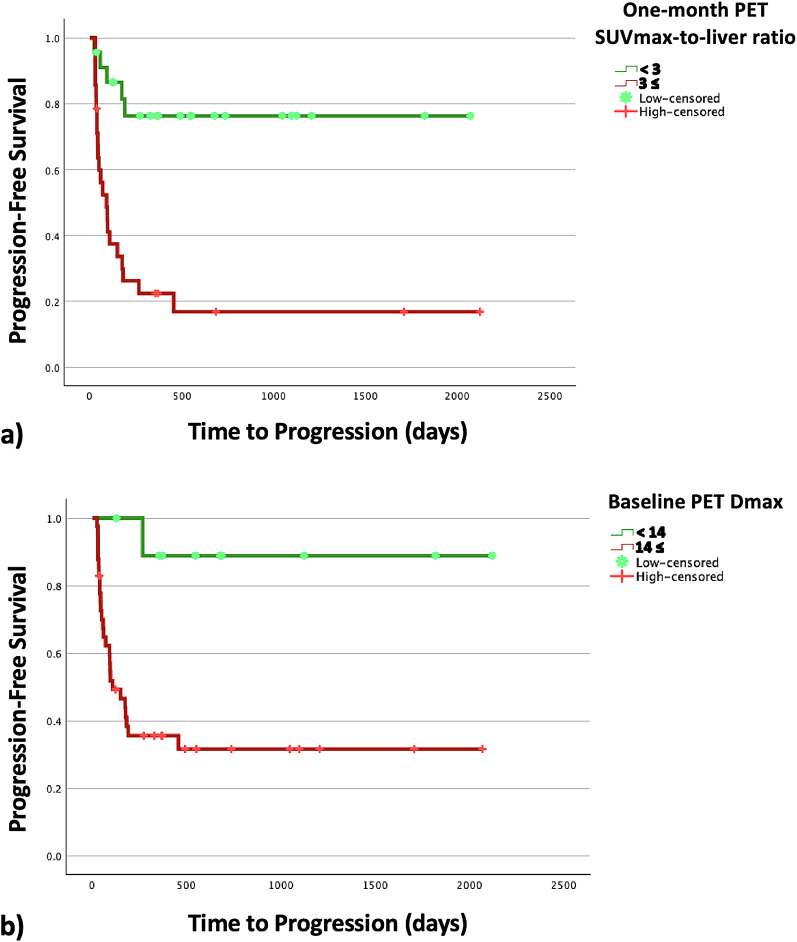
Kaplan-Meier curves for (**a**) one-month post-treatment SUVmax-to-liver ratio and (**b**) baseline Dmax to predict progression-free survival

Thus, the one-month post-treatment SUVmax-to-liver ratio and baseline Dmax were the two variables that could retain their significance in the multivariate analysis, being independent predictors of PFS. The Kaplan-Meier curves for these two parameters to predict PFS are shown in Fig. 3.

### Overall survival

Univariate analysis results can be found in Table 5. From the significant variables and after removing high-co-linear parameters, we entered the following parameters into the multivariate Cox analysis (Table 6): serum LDH (cut-off = 250), delta SUVmean-to-liver ratio (cut-off = 0), delta percentage TLG (cut-off = -50%), and one-month post-treatment Dmax (cut-off = 60).

**Fig. 4:**
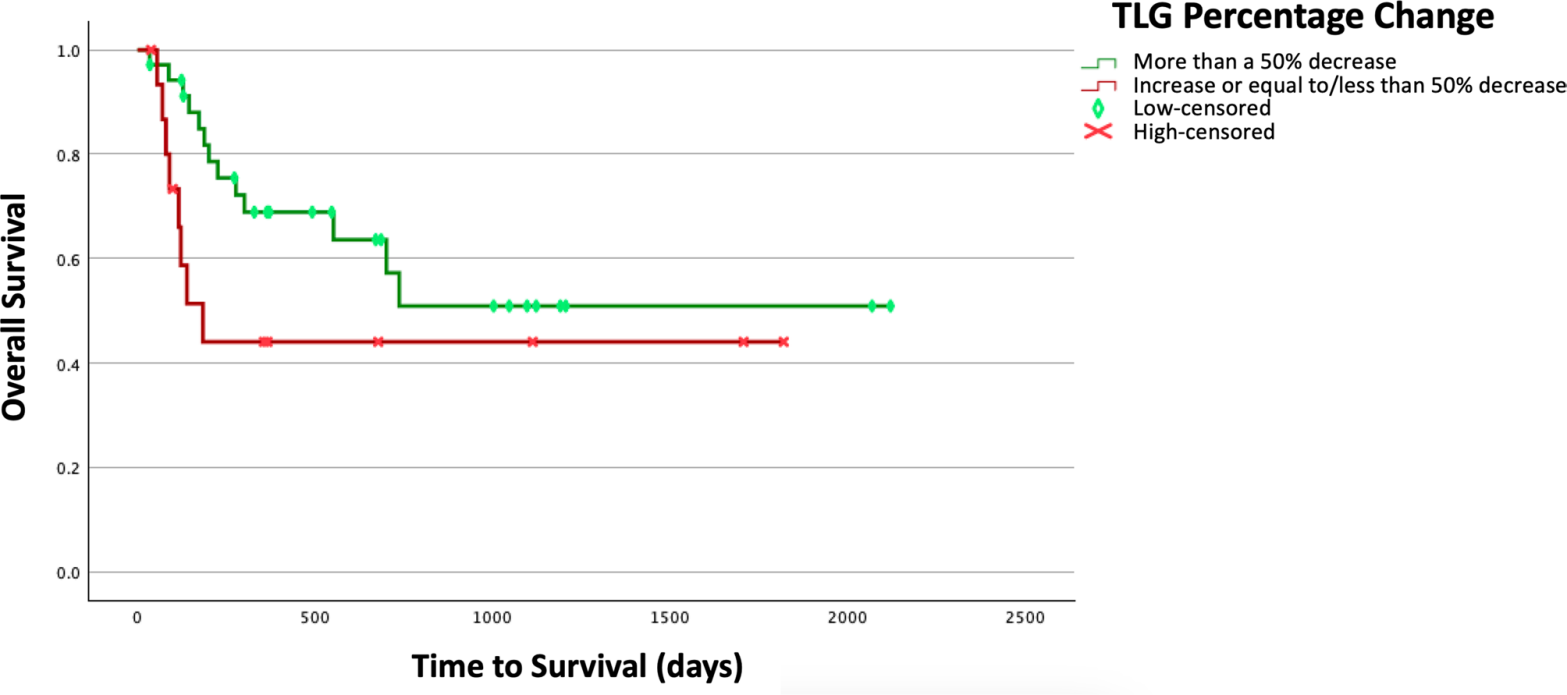
Kaplan-Meier curves for delta percentage TLG to predict overall survival

Thus, the delta percentage TLG was the only parameter that could retain its significance in the multivariate analysis, being an independent predictor of OS. The Kaplan-Meier curves for delta percentage TLG in predicting OS are shown in Fig. 4.

## Discussion

In this retrospective study, we investigated the value of ^18^F-FDG PET/CT findings one month after CAR-T in DLBCL patients. Overall, more than one-fourth of patients experienced a complete metabolic response to treatment one month after infusion, though having significant residual disease on baseline pre-infusion ^18^F-FDG PET/CT. Our findings support the value of early ^18^F-FDG PET/CT imaging in terms of measurements since significant changes were observed compared to the baseline assessment. Specifically, SUVmax, SUVpeak, SUVmax-to-Liver ratio and MTV values were significantly lower in the one-month post-treatment ^18^F-FDG PET/CT.

Moreover, performing ^18^F-FDG PET/CT at both time points was shown to be of benefit for patient prognostication. In terms of PFS, there was a wide variety of clinical and ^18^F-FDG PET/CT-derived parameters that showed statistical significance in the univariate analysis. In the multivariate assessment, the one-month post-treatment SUVmax-to-liver ratio (HR = 5.21) and baseline Dmax (HR = 13.08) retained their significance, showing independent prognostication. For OS prediction, only the delta TLG (HR = 4.37) remained significant in the multivariate evaluation, being an independent prognostic factor. Thus, in summary, the baseline extent of the disease, one-month post-treatment metabolic avidity, and changes in the metabolic burden were significant predictors of patient survival. Having high disease extent and high uptake ratio were indicative of poor PFS, and an increase or less than a 50% decrease in the metabolic burden from baseline could predict poor OS. Noteworthy, among our studied clinical factors, serum LDH was a significant predictor of both PFS and OS in the univariate assessment, but could not retain its significance when evaluated alongside ^18^F-FDG PET/CT-derived parameters, underscoring the value of ^18^F-FDG PET/CT in independent patient prognostication.

Our evaluation adds new information to the currently available body of literature. Several studies evaluated partly similar scenarios, though those did not evaluate DLBCL-only patients, or studied only limited sets of ^18^F-FDG PET/CT parameters. Overall, comparable literature is scarce. Al Zaki et al. investigated a large population of 204 patients one month after treatment and reported a complete response rate of 50%, which seems to be significantly higher than what we witnessed (28%) [13]. In this regard, it should be noted that since their study was not limited to DLBCL patients, a precise head-to-head comparison is not possible. Thus, their reported higher response rate may be related to the better-responding lymphoma histologic subtypes. Having said that, similar to us, they found that achieving a complete response within one month could not predict patients’ survival outcomes reliably. Thus, we need advanced ^18^F-FDG PET/CT parameters for precise risk assessment in this patient population.

Later, Lufti et al. studied a small population of 28 large B-cell lymphoma patients, 38% of whom did not have DLBCL. They showed that a lower SUVmax on one-month post-treatment ^18^F-FDG PET/CT and a lower MTV at both baseline and one-month post-treatment ^18^F-FDG PET/CT could significantly predict better patient survival, considering both PFS and OS [15]. Their findings were somehow comparable to our findings. We also found that one-month post-treatment uptake and metabolic burden are significant predictors of patient survival. However, uptake ratio was shown to be a better predictor of survival than uptake itself, which the former was not evaluated in their study. Also, we showed that if a patient shows a 50% or more decrease in TLG, and not the volume (MTV) alone, overall survival would be predicted more reliably. Notably, Lufti et al. did not measure percentage changes for TLG in their study and they only worked on the percentage changes in MTV.

In another study, with 57% DLBCL patients (overall population = 69 patients), Breen et al. also mentioned that high SUVmax and MTV one month post-treatment were significantly associated with disease progression and patient death [12]. Again, they did not investigate different variations of uptake calculation, whereas we found that the SUV-to-background ratio could be a stronger predictor compared to SUVmax alone. Also, as mentioned earlier, it seems the total metabolic burden calculated as TLG may be a better representative of patients’ metabolic burden than the volume alone (MTV), considering both the volume and avidity of the residual disease. Furthermore, Breen et al. did not opt for multivariate analysis, which, in our opinion, is statistically more robust in terms of pointing out the most beneficial parameters that can stand out among the various possibly extractable ^18^F-FDG PET/CT parameters. We found that the percentage change in TLG from the baseline was the most significant predictor of OS, being the only independent predictor in the multivariate analysis. Also, the one-month post-treatment SUVmax-to-background ratio and baseline Dmax could stand out in the multivariate assessment to predict PFS, showing their robustness in independent prognostication.

Most recently, Gui et al. studied a comparable investigation to ours. They included 38 patients with DLBCL who underwent both baseline and one-month post-treatment ^18^F-FDG PET/CTs [20]. They reported that higher SUVmax and TLG one month after CAR-T would predict shorter PFS. For OS, they found that higher SUVmax one month after therapy and SUVmax percentage change from the baseline were the strongest predictors. However, the drawback in their analysis was that they included both MTV and TLG in their multivariate analysis which made them insignificant due to the known high collinearity of these two parameters. Our methodology was designed to prevent this bias by including the more robust parameter of each group in the multivariate assessment to reach a more reliable assessment in terms of reporting the strongest predictors. Having said that and putting the differences in statistical analyses aside, it seems the main parameters of ^18^F-FDG PET/CT, including uptake value (SUVmax and its ratio) and metabolic burden (MTV and TLG), can significantly predict patient survival, and both baseline and one-month post-treatment scans are required for the most precise assessment. This was also supported by another recent study, in which authors showed that high SUVmax, MTV, and TLG one month after CAR-T therapy were significantly associated with poorer patient survival [21]. Moreover, Leithner et al. showed that in their large population of 180 LBCL patients, higher SUVmax on the pre-infusion ^18^F-FDG PET/CT was associated with poor response to treatment [22]. Also, MTV was the key predictor of survival in their study population.

Lastly, it should be noted that none of the above-mentioned studies investigated the value of the extent of disease measured as Dmax, which can be considered a novelty in our study. We showed that a high baseline Dmax, representative of more extensive disease, would predict an earlier progression during follow-up. Dmax can show another aspect of disease burden in the body, different from the volumetric burden of the disease, by depicting the furthest reach of the lymphoma in the body. This may help clinicians to identify high-risk patients at baseline using a single independent parameter which can be of great benefit for risk assessment. It was previously reported that Dmax at the time of diagnosis is an independent predictor of predicting time to progression, PFS and OS in DLBCL patients, but for newly diagnosed cases and not in the context of CAR-T therapy [23, 24].

Our study has several limitations. The most prominent one was our small patient population. However, contrary to the abovementioned investigations, we limited ourselves to only patients with DLBCL to increase our study’s homogeneity and provide DLBCL literature with a more dedicated investigation. The other major drawback was regarding patient follow-up. We could not follow all patients until disease progression/death documentation, and this could have an impact on our survival analyses. Moreover, measuring different parameters on ^18^F-FDG PET/CT images may be operator-dependent to some extent. However, we used well-established, dedicated software for this purpose which has been used for other publications several times before. Lastly, statistical cut-offs for the studied parameters were defined based on our population characteristics, which may make them subject to change in future studies.

In conclusion, early post-treatment ^18^F-FDG PET/CT can provide valuable prognostic information regarding patient survival in DLBCL patients receiving CAR-T therapy. The most significant predictors of patient outcomes would be the baseline extent of the disease, one-month post-treatment uptake ratio, and changes in the metabolic burden from baseline. High one-month post-treatment SUVmax-to-liver ratio and baseline Dmax were independent predictors of short PFS, and an increase or low interval decrease in TLG from baseline could indicate patients’ poor OS.

## Data Availability

No datasets were generated or analysed during the current study.
